# Infarct evolution in man studied in patients with first-time coronary occlusion in comparison to different species - implications for assessment of myocardial salvage

**DOI:** 10.1186/1532-429X-11-38

**Published:** 2009-09-23

**Authors:** Erik Hedström, Henrik Engblom, Fredrik Frogner, Karin Åström-Olsson, Hans Öhlin, Stefan Jovinge, Håkan Arheden

**Affiliations:** 1 Department of Clinical Physiology, Lund University Hospital, SE-221 85 Lund, Sweden; 2 Department of Cardiology, Lund University Hospital, SE-221 85 Lund, Sweden; 3 Department of Cardiology, Sahlgrenska Hospital, SE-413 45 Gothenburg, Sweden

## Abstract

**Background:**

The time course of infarct evolution, i.e. how fast myocardial infarction (MI) develops during coronary artery occlusion, is well known for several species, whereas no direct evidence exists on the evolution of MI size normalized to myocardium at risk (MaR) in man. Despite the lack of direct evidence, current literature often refers to the "golden hour" as the time during which myocardial salvage can be accomplished by reperfusion therapy. Therefore, the aim of the present study was to investigate how duration of myocardial ischemia affects infarct evolution in man in relation to previous animal data. Consecutive patients with clinical signs of acute myocardial ischemia were screened and considered for enrollment. Particular care was taken to assure uniformity of the patients enrolled with regard to old MI, success of revascularization, collateral flow, release of biochemical markers prior to intervention etc. Sixteen patients were ultimately included in the study. Myocardium at risk was assessed acutely by acute Myocardial Perfusion Single photon emission computed tomography (MPS) and by T2 imaging (T2-STIR) cardiovascular magnetic resonance (CMR) after one week in 10 of the 16 patients. Infarct size was measured by late gadolinium enhancement (LGE) at one week.

**Results:**

The time to reach 50% MI of the MaR (T_50_) was significantly shorter in pigs (37 min), rats (41 min) and dogs (181 min) compared to humans (288 min). There was no significant difference in T_50 _when using MPS compared to T2-STIR (p = 0.53) for assessment of MaR (288 ± 23 min vs 310 ± 22 min, T_50 _± standard error). The transmural extent of MI increased progressively as the duration of ischemia increased (R^2 ^= 0.56, p < 0.001).

**Conclusion:**

This is the first study to provide direct evidence of the time course of acute myocardial infarct evolution in relation to MaR in man with first-time MI. Infarct evolution in man is significantly slower than in pigs, rats and dogs. Furthermore, infarct evolution assessments in man are similar when using MPS acutely and T2-STIR one week later for determination of MaR, which significantly facilitates future clinical trials of cardioprotective therapies in acute coronary syndrome by the use of CMR.

## Background

Final MI size is an important determinant of prognosis in patients with acute coronary syndrome [1,2]. A major determinant of final MI size is duration of ischemia [3], which has been shown to affect patient outcome [2,4,5]. Acute ischemic heart disease leads by necessity to the consequences of chronic ischemic heart disease unless the acute event is treated by revascularization within "the golden hour". The golden hour, however, is mainly derived from retrospective uncontrolled studies of vast patient populations using indirect techniques to assess MI size, and may at best constitute a rough estimate of the dependence on time for infarct evolution in man. Whereas the time course of infarct evolution is well known for several species [3,6-11], no direct evidence exists on the evolution of MI size normalized to MaR in man. Thus, our understanding of infarct biology is to a large extent based on experimental studies and little is known from studies in man. This is mainly because accurate measurement of MI size *in vivo *has been difficult to obtain in humans. Indirect methods such as ejection fraction, biochemical markers of myocardial injury, ECG, echocardiography, or Myocardial Perfusion Single photon emission computed tomography (MPS) have previously been used to estimate MI size. With the introduction of late gadolinium enhancement (LGE) cardiovascular magnetic resonance (CMR), it is now possible to differentiate between viable and non-viable myocardium, and thus assessing MI size with high accuracy *in vivo *[12]. It has been shown in both dogs [13] and humans [14] that absolute MI size, or MI size normalized to left ventricular mass, shows no major relation to duration of ischemia. However, in order to determine how duration of ischemia affects MI size, it is essential to relate MI size to the amount of myocardium subjected to ischemia, i.e. the MaR.

Therefore, the primary aim of the present study was to investigate how duration of ischemia affects myocardial infarct evolution in man using automatically quantified MPS defects and T2-weighted double inversion blood suppressed turbo spin echo sequence (T2-STIR) for MaR and automatically quantified MI size using LGE in patients with first-time MI. A secondary aim was to compare these findings with results from previous experimental animal studies.

## Methods

### Study population

The protocol and procedures were approved by the local research ethics committee. All patients gave their written informed consent to participate. More than 700 consecutive patients with clinical signs of acute myocardial ischemia were screened and considered for enrollment. To be able to compare the results from the present study with prior animal studies, strict inclusion criteria were applied to resemble an experimental setting as much as possible. Therefore, to be considered for inclusion, patients should have no history of old MI, a single occluded artery followed by successful revascularization by primary percutaneous coronary intervention (pPCI) (TIMI grade 3 flow) and absence of gross collateral flow by angiography. Furthermore, patients should not have creatine kinase MB (CKMB) or cardiac troponin T (cTnT) levels above clinical reference values of MI before pPCI to minimize the influence of temporary opening of the occluded coronary artery on final MI size [15].

Duration of ischemia was defined as time from onset of chest pain, as reported by the patient, to opening of the occluded coronary artery by pPCI. To decrease the risk of including patients with false long duration of persistent ischemia, the acuteness of the ischemia was also assessed from the pre-PCI ECG using the previously described Anderson-Wilkins (AW) electrocardiographic acuteness score [16,17]. In short, an AW score between 1 and 4 was designated to each patient, where 1 indicates the longest and 4 indicates the shortest duration of persistent ischemia. Presence of Q waves lower the AW score, whereas tall T waves increase the AW score. Patients were excluded from the analysis of time course of infarct evolution if they had more than three hours between pain onset and pPCI and an AW score of 3 or higher. The AW acuteness score was performed by an experienced electrocardiographer blinded to all other data.

### Study protocol

For determination of MaR, ^99m^Tc tetrofosmin was injected approximately 5 minutes prior to opening of the occluded artery by pPCI, and then the patients underwent MPS within 3 hours. After the intervention the patients were transferred to the coronary care unit for conventional therapy, and had an uncomplicated clinical course between the pPCI and the CMR examination performed one week after admission for determination of MI size and, for some of the patients, also MaR by T2-STIR imaging.

### Myocardial perfusion SPECT

Patients were injected with 500-700 MBq ^99m^Tc tetrofosmin (Amersham Health, Buckinghamshire, UK), depending on bodyweight. The ECG-gated MPS was performed according to the standard clinical protocol at rest, using a dual head-camera, either ADAC Vertex (Milpitas, California, USA) or a cardiac dedicated GE Ventri (GE Healthcare, Buckinghamshire, UK). The patients were placed in supine position and imaged in steps of 5.6 degrees using a 64 × 64 matrix with a pixel size of 5.02 mm for the ADAC camera and a pixel size of 6.4 mm for the GE Ventri camera. Image acquisition time was approximately 15 minutes. Short- and long-axis images gated to the ECG were reconstructed using a commercial application (AutoSpect+InStill™ 6.0, ADAC, Milpitas, California, USA). Analysis of the perfusion defect for MaR was performed using a freely available in-house developed segmentation software (Segment v1.702; ). The automatic segmentation finds the centreline through the left ventricular wall and identifies the endo- and epicardium based on an individually estimated wall thickness and signal intensity values within the image [18]. Manual adjustment of the automatic delineation was sometimes required in the left ventricular outflow region. The perfusion defect was determined by an in-house developed automated algorithm that considers myocardium with < 55% of normal counts, taking into account the different coronary perfusion territories, as being subject to ischemia (Soneson H, Engblom H, Hedstrom E, Bouvier F, Sörensson P, Pernow J, Arheden H, Heiberg E: **A novel automatic method for quantification of myocardium at risk from myocardial perfusion SPECT in patients with acute coronary occlusion**. Submitted). Myocardium at risk was quantified as % of the left ventricle.

### CMR

One week after admission patients were imaged in the supine position using either a 1.5 T system (Magnetom Vision, Siemens, Erlangen, Germany) with a CP body array coil or a 1.5 T system (Philips Intera CV, Philips, Best, the Netherlands) with a cardiac synergy coil. Initial scout images were acquired to locate the heart and T2-weighted double inversion blood suppressed turbo spin echo sequence (T2-STIR) was employed in 10 patients to depict MaR as previously described [19]. T2-STIR images were acquired in the short-axis view, covering the left ventricle from the base to apex. Image parameters for T2-STIR were: echo time of 43 ms (Siemens), or 100 ms (Philips); repetition time, 2 heart beats; number of averages, 2; inversion time of 180 ms; image resolution of 1.5 × 1.5 × 10 mm (Siemens) or 1.5 × 1.5 × 8 mm with a slice gap of 2 mm (Philips). When acquiring images with the cardiac synergy coil, parallel imaging with SENSE = 1 was used to minimize signal inhomogeneities due to differences in coil sensitivity. The MaR, defined as the region with increased signal intensity in the short-axis T2-STIR images [19], was manually delineated by two observers blinded to all other data. A third independent observer was consulted for consensus to enable comparison with infarct evolution determined by MPS for assessment of MaR.

For infarct vizualisation, short- and long-axis LGE images were acquired typically 20-30 minutes after administration of 0.2 mmol per kilogram of body weight of an extracellular gadolinium-based contrast agent (*gadoteric acid*, Gd-DOTA, Guerbet, Gothia Medical AB, Billdal, Sweden). Acquisition was performed during breath-hold, using an ECG-triggered segmented inversion-recovery gradient recalled echo (IR-GRE) sequence. Typical IR-GRE sequence parameters were: echo time/repetition time of 4/8 ms; image resolution of 1.4 × 1.4 × 10 mm and flip angle of 25° with acquisition every other heartbeat for the Siemens scanner and echo time/repetition time of 1.2/3.9 ms; image resolution of 1.5 × 1.5 × 8 mm and flip angle of 15° with acquisition every heartbeat for the Philips scanner. The inversion time of typically 200-350 ms was manually adjusted to null the signal from remote myocardium.

Infarct quantification was performed on the short-axis LGE images using the same software as for the MPS images (Segment, v.1.702; ) by employing a previously described automatic algorithm for MI border detection [20]. The MI borders were visually controlled and adjusted if the computer algorithm was obviously wrong. Infarct transmurality was assessed using a centerline approach, assessing the radial extent of hyperenhancement at each 4.5° from the center of each short-axis slice. Infarct size was expressed as % of the left ventricle. Infarct size by LGE was then normalized to MaR by MPS and T2-STIR.

### Comparison to previous animal studies

Species other than humans are frequently used in phase I-II experiments, and canine work regarding myocardial cell death beginning 30 minutes after occlusion is often quoted. Therefore, it is important to compare the findings from the present study in man with earlier results from animal studies. For this purpose, analysis of MI size normalized to MaR for rats [8,11], pigs [6,9], and dogs [3,7,9,10] was performed and compared with the human data in the present study.

### Statistical analysis

The MI data as a function of duration of ischemia was modelled by a simple function that embodies the following features: After a short ischemic period the myocardium is inviolate; after such a period the MI/MaR ratio rises in a sigmoid fashion to a plateau level. The model was constrained to the following form: MI/MaR at t minutes after occlusion = 100/(1+((T_50_-15)/(t-15))^2^), which focuses on the time to 50% MI/MaR, T_50_. This function was fitted by non-linear least-squares technique, and error estimates on T_50 _obtained by first-order error propagation [21]. These error estimates were calculated using data from each species individually as well as using pooled data. The former were used to obtain 95% confidence levels for visual inspection, and the latter for multiple t-tests with Sidak correction to test the separation between species and between MPS and T2-STIR in terms of T_50_.

The relationship between the maximum infarct transmurality and duration of ischemia was analyzed by the following sigmoidal model: Infarct transmurality after t minutes = 100/(1+(Tr_50_/x)^z^), where Tr_50 _is time to 50% transmurality, x the duration of ischemia and z the speed at which MI transmurality progresses with increasing duration of ischemia. This function was fitted by non-linear least-squares technique. The critical value of Student's t distribution was used to derive the p-value for the correlation between the function's prediction and measured values. P-values < 0.05 were considered to indicate statistical significance.

## Results

### Patient characteristics

Eighteen patients (17 men; 41-83 years) were prospectively enrolled. Patient characteristics are presented in Table 1. Duration of ischemia, AW score, MaR by MPS, and MI size by LGE, are presented in Table 2. All patients had ST elevation suggestive of acute myocardial ischemia and underwent pPCI with stenting and received glycoprotein (GP) IIb/IIIa inhibitor resulting in TIMI grade 3 flow. Two patients (#4 and #11, Table 2) reported more than 3 hours between pain onset and opening of the occluded artery and had an AW score of 3 or higher. Therefore, these two patients were excluded from further analysis. One patient had 387 min between pain onset and opening of the occluded artery. This patient had positive biomarkers (CKMB 108 μg/l and cTnT 0.37 μg/l) before pPCI. However, since the artery was occluded at the time of angiography, and that it is reasonable to believe that biomarkers start to leak into the blood after more than 6 hours of occlusion, this patient was included in the study.

**Table 1:** Patient characteristics

Age (years)	67 (41 - 83)
Male	17 (94%)
Culprit coronary artery	
LAD	8 (44%)
LCx	1 (6%)
RCA	9 (50%)
TIMI grade 0 flow before pPCI	18 (100%)
TIMI grade 3 flow after pPCI	18 (100%)
Duration of ischemia (min)	158 (60 - 387)
MaR (% of left ventricle by MPS)	23 (5 - 48)
Infarct size (% of left ventricle)	7 (0 - 21)
Relative infarct size (% of MaR by MPS)	22 (0 - 54)
Previous angina pectoris	2 (11%)
Former smoker	9 (50%)
Active smoker	6 (33%)
ACE inhibitor	1 (6%)
Acetyl salicylic acid	2 (11%)
Angiotensin-II receptor blocker	1 (6%)
Beta blocker	0 (0%)
Calcium channel blocker	1 (6%)
Diuretic	2 (11%)
Statin	1 (6%)
Oral anti-diabetic	1 (6%)
Insulin	3 (17%)

Values as median (range) or frequency (%). ACE = angiotensin converting enzyme, LAD = left anterior descending coronary artery, LCx = left circumflex coronary artery, MaR = myocardium at risk, MPS = myocardial perfusion single photon emission computed tomography, pPCI = primary percutaneous coronary intervention, Rca = right coronary artery, TIMI = thrombolysis in myocardial infarction.

**Table 2:** Duration of ischemia and corresponding AW-score, myocardium at risk and MI size.

**Case**	**Duration of ischemia (min)**	**AW score**	**MaR (% by SPECT)**	**MI (%)**	**MI/MaR (% by SPECT)**
1	190	-^†^	37	11	31
2	60	3.1	- *	0	0
3	145	2.2	24	4	16
4	205	3.7	58	0	0
5	135	2.8	21	2	8
6	175	-^†^	5	2	40
7	80	2.8	32	9	29
8	245	2.7	25	8	31
9	110	3.0	48	2	4
10	135	4.0	24	2	7
11	240	3.0	71	7	10
12	150	1.0	29	11	37
13	96	3.2	34	0	0
14	195	-^†^	37	13	35
15	120	2.4	48	11	23
16	387	1.0	38	21	54
17	104	4.0	19	3	15
18	196	2.0	34	9	27

AW = Anderson-Wilkins score, MaR = myocardium at risk by SPECT. Infarct = infarct size by LGE.

* Please see Results for explanation, ^† ^AW scoring not possible.

### Infarct size and myocardium at risk

The MI size by LGE was 7% (range 0-21%) of the left ventricle. The MaR was 30% (range 5-48%) of left ventricle as assessed by MPS and 32% (23-43%) of the left ventricle on 10 of the 16 patients also assessed by T2-STIR. Interobserver variability for determination of MaR by T2-STIR was 1 ± 5 percentage units. Posterior wall signal loss and increased signal intensity in the blood pool at the apical parts of the left ventricule due to stagnant blood was frequently seen in the T2-STIR short-axis images making the determination of the endocardial and epicardial borders challenging. This was solved by guidance from the short-axis cine images in the corresponding level.

### Infarct evolution in relation to duration of ischemia

**Figure 1:**
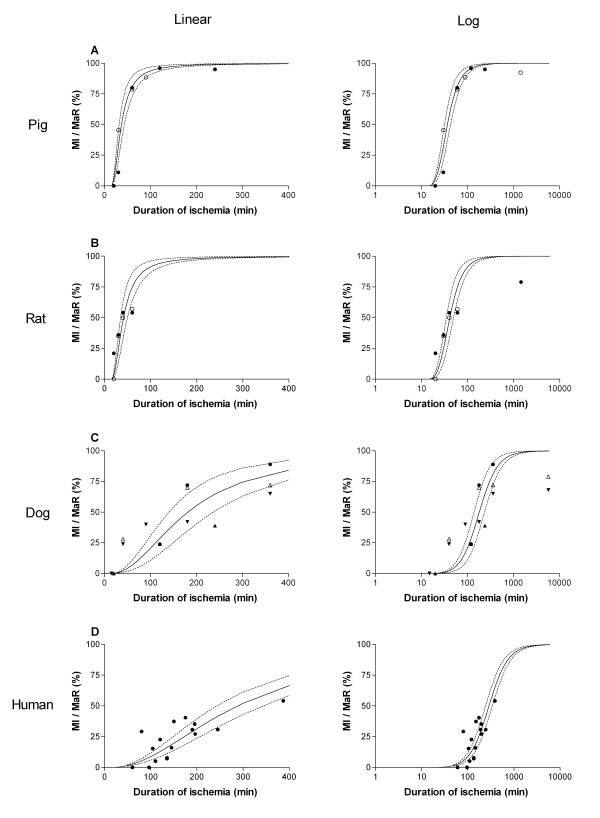
**Myocardial infarct size in relation to myocardium at risk with respect to duration of ischemia in different species**. A linear and logarithmic time scale is presented in the left and right panel, respectively. **A: **Data from previous studies in pigs (black circle = Fujiwara et al [9], white circle = Näslund et al [6]. **B: **Data from previous studies in rats (black circle = Hale et al [8], white circle Arheden et al [11]). **C: **Data from previous studies in dogs (white triangle = Reimer et al [3], black circle = Kloner et al [7], upward black triangle = Fujiwara et al [9], downward black triangle = Reimer et al [10]). **D: **Human data from the present study. The dashed lines represent the 95% confidence intervals. MaR = myocardium at risk; MI = myocardial infarction.

**Figure 2:**
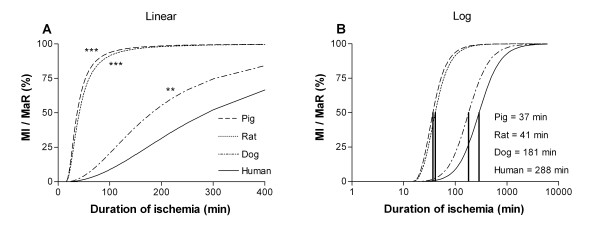
**Infarct progression for different species**. Comparison of the infarct progression slope (**A**) and the time to reach 50% MI of the MaR (**B**) for the different species. There was a significantly slower infarct evolution in man compared to pigs, rats and dogs. Consequently, the time to reach 50% MI of the MaR was longer for humans compared to pigs, rats and dogs. *** = p < 0.001; ** = p < 0.01; MaR = myocardium at risk; MI = myocardial infarction.

**Figure 3:**
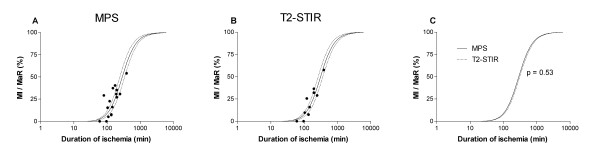
**Infarct evolution in man: MPS vs T2-STIR for myocardium at risk**. Infarct evolution when using (**A**) MPS and (**B**) T2-STIR for assessment of MaR. The dashed lines represent the 95% confidence intervals in **A **and **B**. There was no significant difference between MPS and T2-STIR for assessing infarct evolution (**C**). MaR = myocardium at risk; MI = myocardial infarction; MPS = myocardial perfusion SPECT; T2-STIR = T2-weighted double inversion blood suppressed turbo spin echo CMR sequence.

Infarct size in relation to MaR was found to increase with duration of ischemia (Figure 1A-D). Figure 2A shows that the infarct evolution in man was considerably slower than infarct evolution in rats (p < 0.001), pigs (p < 0.001) and dogs (p < 0.01). The time to reach 50% MI of the MaR (T_50_) was considerably shorter for pigs and rats (37 and 41 min, respectively) compared to dogs and humans (181 and 288 min, respectively) (Figure 2B). There was no significant difference (p = 0.53) in T_50 _for the humans when using MPS (288 ± 23 min) compared to T2-STIR (310 ± 22 min) for assessment of MaR (Figure 3).

In one patient (# 2, Table 2) with occlusion of the right coronary artery, the MaR was not assessed due to lack of MPS isotope. In this patient, biochemical markers of cardiac injury were below reference level before pPCI and rose to maximally 55 μg/l (CKMB) and 2.1 μg/l (cTnT) after reperfusion. These findings indicate occlusion before pPCI, successful reperfusion, and infarcted myocytes. This patient had no MI visible by LGE and was therefore included in the analyses as 0% MI of the MaR, although no measurement of MaR was obtained.

**Figure 4:**
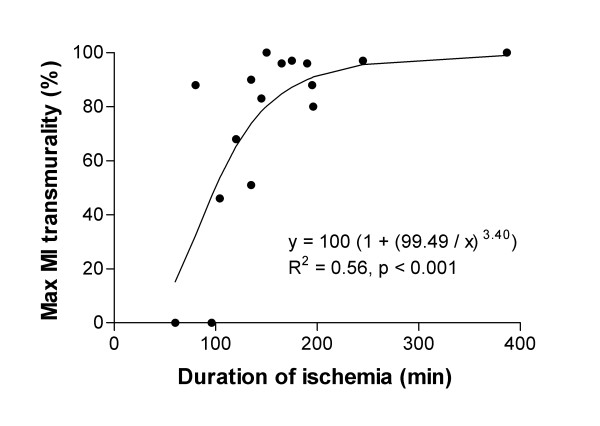
**Infarct transmurality in relation to duration of ischemia in man**. As duration of ischemia increased the transmural extent of MI increased progressively. MI = myocardial infarction.

Figure 4 shows that maximum MI transmurality increased with increased duration of ischemia (R^2 ^= 0.56; *t *= 4.3 corresponding to p < 0.001). The time to reach 50% transmurality (Tr_50_) was 99 min.

**Figure 5:**
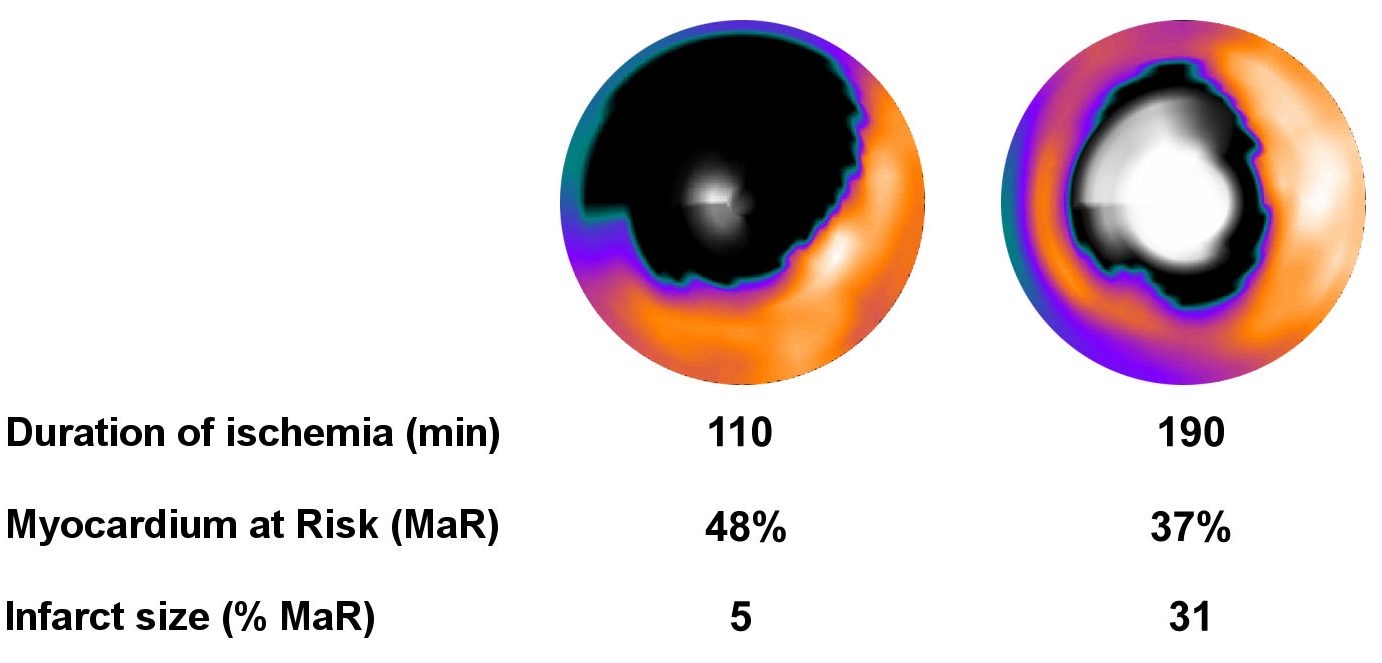
**Representative cases**. Fusion of polar plots from MPS acquired acutely and LGE images acquired one week after the acute MI in two patients subjected to ischemia for 110 and 190 minutes, respectively. The MPS polar plots indicate MaR (black) in the otherwise well perfused myocardium (yellow). The center of the polar plot represents the left ventricular apex and the periphery represents the basal parts of the left ventricle. Final MI size by LGE is shown in white, where brightness indicates MI transmurality from 0% (black) to 100% (white). LGE = late gadolinium enhancement; LV = left ventricle; MaR = myocardium at risk.

Two examples of the infarct evolution normalized to MaR in patients are shown in Figure 5.

## Discussion

This is the first study to provide direct evidence of the time course of acute myocardial infarct evolution in relation to MaR in man with first-time MI. Infarct evolution in man is significantly slower than in pigs, rats and dogs. Furthermore, infarct evolution assessments in man are similar when using MPS acutely and T2-STIR one week later for determination of MaR, which significantly facilitates future clinical trials of cardioprotective therapies in acute coronary syndrome by the use of CMR.

In animal studies of the relationship between duration of ischemia and MI size in relation to the MaR, the time of coronary occlusion is very well defined. Also, the experimental setting enables controlling that the coronary artery remains occluded for the entire duration of ischemia. Unlike the experimental setting, it is impossible to define the exact time of occlusion in patients suffering from acute coronary occlusion. Furthermore, spontaneous reperfusion and re-occlusion may occur between pain onset and opening of the occluded artery by pPCI. In humans, factors such as preinfarction angina, stuttering occlusion/reperfusion, collateral blood flow, and many other factors complicate precise determination of duration of ischemia. In the present study, strict inclusion criteria were used to limit analysis to patients with first-time MI, single occluded artery at pPCI (TIMI 0), no angiographic evidence of collaterals to the infarct-related territory, exclusion of patients with elevated levels of biochemical markers prior to pPCI, and patients where the reported duration of ischemia corresponded with the AW acuteness score that relates ECG findings to the duration of occlusion. If indeed spontaneous reperfusion and re-occlusion do occur, ischemic preconditioning may also explain why no or only a small part of the MaR is eventually infarcted. Thus many of these factors are critical when interpreting the results.

Patients with visible collateral flow by angiography were excluded from the present study to exclude patients with prior ischemic heart disease. Small collaterals, not visible by angiography, may still be present in patients included in the present study. However, such a collateral flow would not affect the measurement of MI size in relation to MaR since the MaR, as assessed by MPS, by nature lacks both native and collateral perfusion.

Late reperfusion has been shown to have a positive effect on outcome after acute MI in man [22,23]. This may partly be explained by spontaneous reperfusion and re-occlusion occurring between pain onset and reperfusion therapy. Thus, the time between pain onset and reperfusion by pPCI might not reflect the actual duration of persistent ischemia. The present study directly demonstrates that timely reperfusion is of importance to limit MI size in humans, but also that late reperfusion in some cases may salvage a significant amount of the MaR.

Currently, MPS is considered the reference technique for quantifying MaR by injecting a perfusion tracer during coronary occlusion followed by imaging shortly after the reperfusion therapy. For assessment of MaR in acute coronary occlusion, this technique requires intravenous injection of a technetium labeled perfusion tracer before the occluded artery is opened and a access to a gamma camera within 3 hours of the injection. Consequently, the logistics for such investigations are complicated and clinically cumbersome [24]. Thus, there is a need for methods that can be used to assess MaR remote to the time of the acute event. It has previously been shown that T2-STIR can be used to quantify MaR remote to the time of artery occlusion in an experimental setting [25,26]. Friedrich et al [27] have shown that T2-STIR can be employed in humans to assess the amount of reversibly injured myocardium after acute MI. Recently, Carlsson et al [19] validated T2-STIR against MPS in humans and showed that the technique can be used to assess MaR one week after an acute coronary occlusion. In the present study, there was indeed no difference in assessment of infarct evolution in man when MaR was assessed acutely by MPS or one week after the acute MI using T2-STIR. Hence, both MI size and MaR and, consequently, myocardial salvage can be assessed at the same CMR image session 1 week after the acute coronary event, without interfering with patient care in the acute situation. This enables evaluation of the efficiency of interventions in acute coronary syndrome and the opportunity to study infarct evolution several days after the acute event. Image quality can, however, be a limitation in assessing MaR by T2-STIR and the image quality of the present study did not allow for automated segmentation. New sequences, however, are continuously developed to overcome this problem [28,29].

### Comparison to animal studies

In order to compare the results from the present study with results from previous animal studies, analysis of MI size normalized to MaR versus duration of ischemia in different species [3,7-11] was performed (Figure 1, 2).

Since the degree of collateral flow is a proven modifier of MI size [30-32], the difference in time course between species could be explained by lack of collaterals in rats and pigs, compared with more extensive collateral interconnections in dogs [33]. It is generally accepted that collaterals are present also in humans, however poorly developed [34,35], and it has been shown in primates that only 20% of MaR is infarcted after 90 minutes of occlusion [36]. This is in the same order of magnitude as the findings in patients in the present study. Therefore, the differences between species could also be explained by different protective mechanisms and different supply and demand of the ischemic myocardium.

The increased MI transmurality with increased duration of ischemia found in the present study has previously been described as the "wavefront phenomenon" in dogs by Reimer and Jennings [3]. In their study 50% transmurality was reached sometime between 40 min and 180 min after coronary occlusion, which is in the same order of magnitude of what was found in the present human study (99 min).

### Limitations of the study

The number of studied patients is small. This was, however, the result of minimizing confounding factors to achieve a study population that was as close to an experimental setting as possible. The included patients thereby yield results that are comparable to experimental animal studies, which was the aim of the study.

Infarct size may also have been influenced by incomplete reperfusion at the myocardial level even though TIMI grade 3 flow was obtained at the time of pPCI in all patients. To increase the probability of successful reperfusion even at the myocardial level, all patients received GPIIb/IIIa inhibitor in conjunction with the pPCI. The GPIIb/IIIa inhibitor has been presented as a promising intervention in combination with PCI for preventing obstruction of microvascular vessels [37].

The time from pain onset to opening of the artery used for estimating the duration of ischemia has inherent limitations as discussed above. However, in our opinion, this study is as close to an experimental setting as possible when studying clinical patients.

The MIs in the present study are modest in size (0-21% of the left ventricle), and extrapolation to larger MIs cannot be performed. The relatively small MI sizes could be related to the relatively short duration of ischemia in the present study. The short duration of ischemia resulted from short transportation distances and the aggressive clinical approach by which the patients are transported by ambulance directly to the catheterization laboratory.

The findings in the present study cannot be generalized to infarct evolution in females, since only one female was included in the study.

## Conclusion

This is the first study to provide direct evidence of the time course of acute myocardial infarct evolution in relation to MaR in man with first-time MI. Infarct evolution in man is significantly slower than in pigs, rats and dogs. Furthermore, infarct evolution assessments in man are similar when using MPS acutely and T2-STIR one week later for determination of MaR, which significantly facilitates future clinical trials of cardioprotective therapies in acute coronary syndrome by the use of CMR.

## Competing interests

The authors declare that they have no competing interests.

## Authors' contributions

EH enrolled the majority of the patients, participated in the study design, participated in data analysis and drafted the manuscript. HE enrolled patients, performed data analysis and finalized the manuscript. FF, KÅO, HÖ, SJ recruited patients from the coronary care unit, were involved in designing the study and drafted the manuscript. HA conceptualized and had the overall responsibility of the study, provided access to the different imaging modalities, participated in the study design and drafted the manuscript. All authors read and approved the final version of the manuscript.
